# Additively Manufactured Composite Lug with Continuous Carbon Fibre Steering Based on Finite Element Analysis

**DOI:** 10.3390/ma15051820

**Published:** 2022-02-28

**Authors:** Chethan Savandaiah, Stefan Sieberer, Georg Steinbichler

**Affiliations:** 1 Biobased Composite and Processes, Wood K Plus-Kompetenzzentrum Holz GmbH, 4040 Linz, Austria; c.savandaiah@wood-kplus.at; 2 Institute of Structural Lightweight Design, Johannes Kepler University Linz, 4040 Linz, Austria; 3 Institute of Injection Moulding and Process Automation, Johannes Kepler University Linz, 4040 Linz, Austria; georg.steinbichler@jku.at

**Keywords:** additive manufacturing, continuous fibre reinforcement, principal strain, optimisation, stiffness and strength analysis, experimental testing, digital image correlation

## Abstract

In this study, the influence of curvilinear fibre reinforcement on the load-carrying capacity of additively manufactured continuous carbon fibre reinforced necked double shear lugs was investigated. A curvilinear fibre placement is descriptive of layers in extrusion-based continuous-fibre-reinforced additive manufacturing with carbon fibres aligned in the directions of principal stress. The alternating layered fibre trajectories follow the maximum and minimum principal stress directions due to axial tension loading derived from two-dimensional finite element analysis (FEA). The digital image correlation was utilised to monitor the strain distribution during the application of tensile load. The 2D FEA data and the tensile test results obtained were comparable, the part strength and the linear approximation of stiffness data variability were minimal and well within the acceptable range. Nondestructive fractography was performed by utilising computed tomography (CT) to analyse the fractured regions of the tensile-tested lug. The CT scanned images aided in deducing the failure phenomenon in layered lugs; process-induced voids and fibre layup undulation were identified as the cause for lug failure.

## 1. Introduction

The behaviour of materials extrusion (MEX) additive manufacturing of short-fibre-reinforced thermoplastic composites had been well studied as processing effects on thermomechanical and morphological properties [1,2]. Several researchers have investigated the influence of fibre length distribution [3,4], process-induced anisotropy [5] and thermal material [6] properties on the functional performance [7] of the parts produced via MEX composite additive manufacturing. The key aspect for the adoption of composite MEX is the light-weight potential of parts and assemblies by functional optimisation of the end-user application [8]. The design considerations for MEX composite structures are based on the multiple layers of fibre-reinforced matrix [9]. The short fibres in each layer are straight and parallel to each other and mostly aligned in the printing direction, causing anisotropic material behaviour [10,11]. Optimising the fibre alignment within each layer to alternating orientation from point to point has not been adopted, albeit due to the nonavailability of necessary commercial tools. Moreover, MEX composite parts are fairly lucrative in terms of overall economics but have poor mechanical and thermal material characteristics [12,13].

The innovative continuous carbon fibre reinforced composite materials in MEX have yet to realise full potential [14,15]. Azarov et al. [16] assessed the complete product development cycle, determining the mechanical properties for the frame material, modelling and the structural analysis of small-scaled autonomous drones. The authors concluded that MEX type processing of continuous fibre composites is a highly promising technology for the manufacturing of lightweight structures for recreational and industrial drones alike. Furthermore, Borowski et al. [17] studied process-induced consolidation of continuous carbon fibre polycarbonate, including the parameterisation of printing temperature to print standard coupons, and performed three-point bending tests to determine the quality of the in situ consolidated MEX coupons. Furthermore, a highly curved test structure was fabricated to determine the limits of the processing with help of computed tomography. Similarly, Dickson et al. [18] evaluated the performance of several MEX-processed carbon, aramid and glass continuous fibre reinforced standard coupons subjected to tension and flexure standard testing. They concluded that the tensile strength of continuous carbon fibre reinforced MEX coupons was up to 6.3 times that of nonreinforced thermoplastic MEX coupons.

Zhu et al. [19] proposed fibre path optimisation to improve the uniaxial tensile load carrying ability of composite laminates with a centred hole using finite element method. The researchers reported a reduction in maximum strength indexes ranging between 19% based on maximum stress criterion and 39% based on Tsai–Wu failure criterion. In addition, Sugiyama et al. [20] and Ferreira et al. [21] proposed optimised variable fibre volume fraction and stiffness composites for curved fibre trajectory along the principal stress direction around the hole. The specimens fabricated with optimised fibre trajectories using continuous carbon fibre additive manufacturing were 1.6 times stronger than conventional linear laminate composites. The use of carbon-fibre-reinforced composite materials in this manner omits its major attribution: optimised strength and stiffness may be aligned in the directions which correspond to the applied loads. In terms of optimisation, Ghiasi et al. [22,23] suggest two distinct design considerations, constant stiffness and variable stiffness. The constant stiffness design terminology in MEX composite additive manufacturing relates to fibre layup with a similar raster angle for the whole fabricated part. However, in variable stiffness design, the optimised fibre paths reinforce the high strain regions, thus eliminating the excess use of fibre reinforcement and simultaneously decreasing the part weight.

The structural design under investigation in this study, a necked double shear lug, contains a geometric discontinuity, a hole, which interposes the fibre continuity and causes a concentration and realignment of stress. The specific issue in this study is to use fibre reinforcement in such a way that the direction of the fibres, or at least some of the fibres, is a function of spatial position in the structure which may lead to steep increases in structural performance. This particular study examines the departure from constant stiffness design use of composite materials in additive manufacturing. The researchers investigated a variable stiffness design approach for the structural part based on principal stress directions fabricated via MEX composite additive manufacturing. In this study, within the variable stiffness design, an optimised elementwise design is considered [24,25]. It creates a distinct orientation for every finite element of a mesh representing a necked double shear lug based on the simulated load conditions. Furthermore, it is postprocessed to derive the final fibre paths for feasible processing in MEX. 

## 2. Materials and Methodology

A commercially available 20 wt.% short carbon fibre (diameter 7 µm, measured weighted average length 220 µm and density 1.72 g·cm^−3^) reinforced polyamide 6 (PA6) filament with a diameter of 1.75 mm was supplied by Prirevo 3D Solutions GmbH, Ried im Traunkreis, Austria, and the material data were taken from the article (see Figure S1). Additionally, the PA6 infused continuous carbon fibre (CCF) with 0.36 mm diameter was purchased from Markforged, Watertown, MA, USA, with fibre volume fraction of approximately 35% [26].

To orientate the fibres best to the principal strain or stress directions in the considered part with fixed geometrical boundary conditions, a numerical approach was applied. First, an isotropic 2D finite element (FE) model was set up in ABAQUS (2017, 3DS, Waltham, MA, USA), assuming quasi-isotropic, linear elastic material behaviour [8]. Quadratic elements are used as they are necessary for the bending part, and nonlinear geometry is used for the stress and strain calculation in FE. Laminate stiffness values are taken from eLamX software (V2.6, TU Dresden, Germany) using 0° stiffness of the CCF (80000 MPa, value taken from in-house test setup) and assuming transversal and shear values. For this initial model, the directions of principal stresses and strains under the presumed test load case were obtained. These data were postprocessed in MATLAB (R2017, MathWorks, Natick, MA, USA) where principal strain orientations were calculated for each element based on the isotropic results (see, e.g., [27] for further details on strain trajectory calculation and potential applications). Principal axis orientations were then fed back to the FE model as individual element material orientations. Using postprocessed data points from MATLAB, alternating layers of minimum and maximum principal stress fibre trajectories were traced within the outline of the part with help of spline command in computer-aided drafting software NanoCAD 5.0. (Nanosoft, Moscow, Russia) A proprietary plugin developed for the drafting software, NanoGcode (v. 0.3.4.41535, Nanosoft, Moscow, Russia), was provided by Anisoprint and was utilised to generate print settings and subsequent G-codes for printing curvilinear fibre paths in Anisoprint A4 Composer 3D printer.

**Figure 1:**
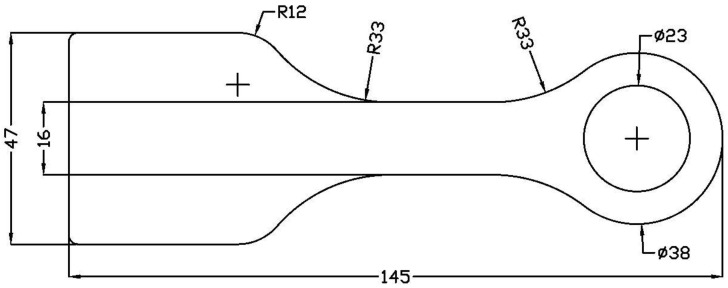
Double sheared lug specimen dimensions (mm).

**Figure 2:**
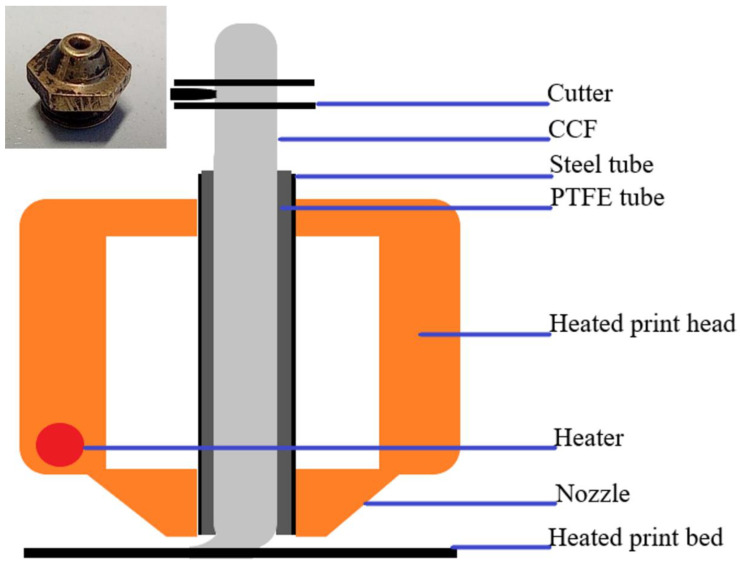
Modified CFC print head from Anisoprint for printing CCF; nozzle in the inset.

The details of print and layer settings are summarised in Table 1, and the specimen dimensions are shown in Figure 1. A slight modification to the existing A4 Composer printing head was required to print the thermoplastic-based CCF. The printer consists of two print heads, a conventional print head for printing pure thermoplastic or filled thermoplastic filament and a composite coextrusion print head, which is analogous to cable coating. The composite coextrusion print head is designed to print proprietary thermoset-based CCF via coating with thermoplastic (as a binder). Hence, a cylindrical stainless steel tube with an internal diameter of 2 mm inlay with polytetrafluorethylene microtubing with an internal diameter of 0.45 mm was used as a thermoplastic CCF feeding tube (Figure 2). The feeding tube acts as a thermal barrier until the nozzle exit, and upon exiting the nozzle, the fibres are uniformly ironed onto the previous layer with help of a tapered nozzle (see Figure 2 inset).

**Figure 3:**
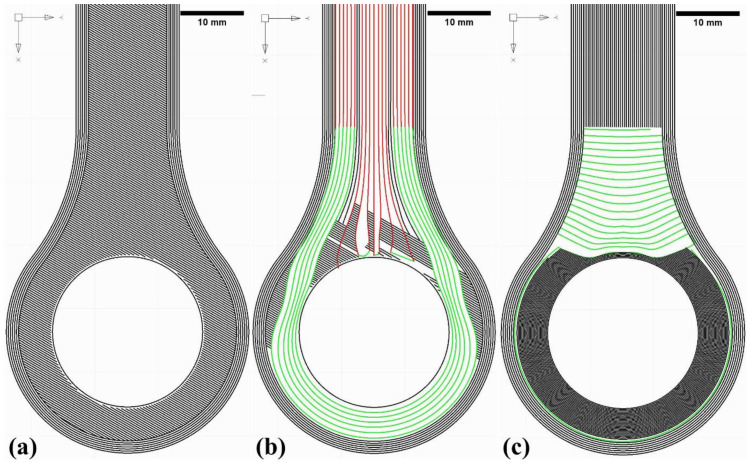
Principal strain trajectories based on the isotropic FE model; green and red coloured splines represent CCF layup, and grey and dark grey colours represent thermoplastic for perimeters and infills. Top and bottom thermoplastic layer (**a**); maximum principal stress direction CCF (**b**), minimum principal stress direction CCF (**c**).

Optimised manufacturing parameters for printing with CCF were utilised to minimise the process-induced defects. The different coloured CCF layup in Figure 3b represents transient printing speed to accommodate the printing accuracy around sharp turns; red colour represents printing at a higher speed compared to the green coloured CCF layup (see Table 1). Moreover, the large gaps observed between the CCF layup in Figure 3b,c are filled with thermoplastic infills to reduce the voids. Furthermore, the CCF layup is a continuous loop, meaning each layer has just one fibre cut at the end of the CCF printing, enabling fibre layup accuracy and reducing the overall print time. The maximum principal strain trajectories are shown in red and green (Figure 3b). Minimum principal strain trajectories are perpendicular to the maximum principal strains and are exemplarily shown in green in the shaft region of the lug (Figure 3c); the dark grey coloured section corresponds to just thermoplastic printing without CCF.

**Figure 4:**
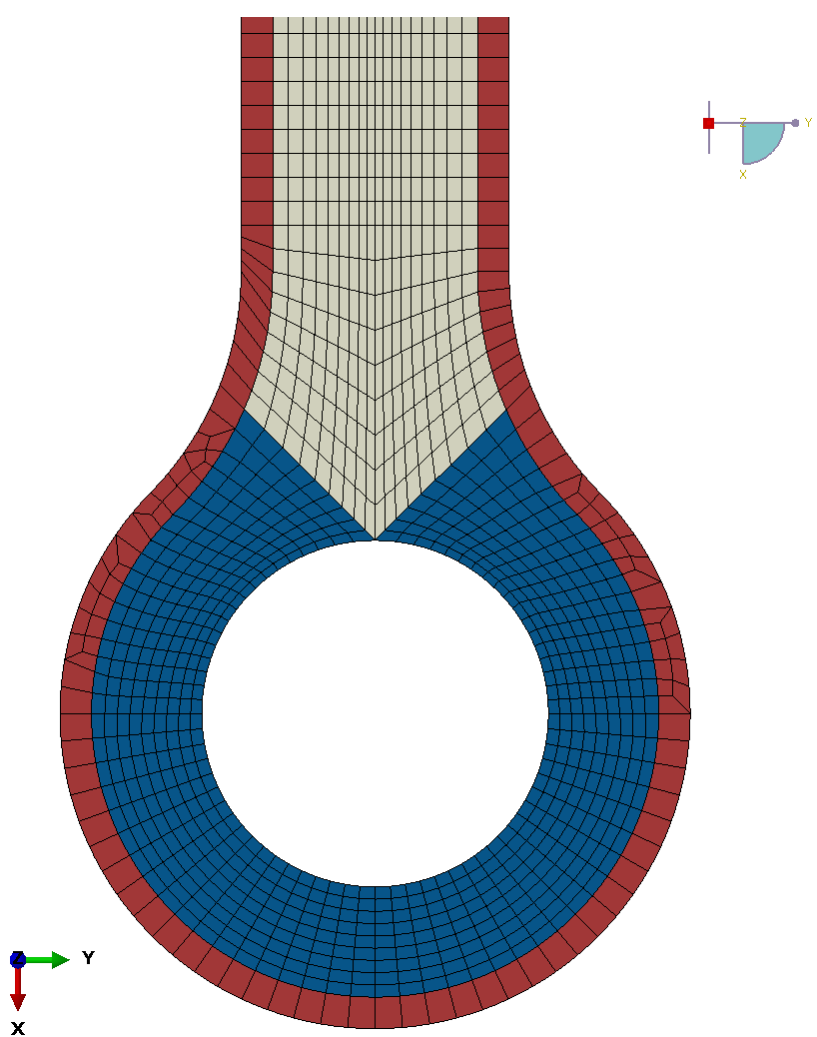
Material and mesh on the orthotropic model.

From the initial results, the print layup was obtained and an updated FE model with orthotropic material properties was set up. Figure 4 shows the mesh and material orientations used in the orthotropic FE model. The outside perimeter (red) has no CCF reinforcements, the region around the lug head (blue) is uniaxial CCF-reinforced in maximum principal strain direction according to the previous results, and the shaft (beige) section is biaxial CCF-reinforced, in both principal strain directions. This modelling reflects the printing restrictions in the lug head and the necessary perimeter. The FE analysis was repeated, and stress and strain data for the part with optimal CCF layup were yielded. Moreover, the curvilinear layup of the specimen corresponds largely but not fully with the material editing in the FE model; however, this discrepancy is small.

A total of four specimens were printed, and the experimental testing was performed on a servohydraulic test rig (Zwick Roell, Ulm, Germany) with a cylinder rated at 100 kN force. The shaft end of the specimen was designed with a flat, wide end (see Figure 1) to be clamped in hydraulic wedge grips. A purpose-built test jig using a steel pin with 23 mm diameter was used to fix the lug eye to the test bed. The test rig was operated by Cubus software (V2.48, Zwick Roell, Ulm, Germany) in displacement mode with a constant rate of 1 mm/min. A Zwick Roell force transducer and the internal displacement-sensor of the cylinder were used for measurement of force and displacement, respectively. Additionally, a Correlated Solutions 3D digital image correlation (DIC) system recorded the surface displacement and calculated the surface strains of the specimens during the tests. Postprocessing of the DIC data was performed in the software Vic 3D 8 (Correlated Solutions, Irmo, SC, USA).

X-ray computed tomography (CT) is used for characterising the fractured specimens. Therefore, scans were conducted on a Nanotom 180 NF (GE Phoenix X-ray, Niskayuna, NY, USA) laboratory CT device. A molybdenum target and a tube voltage of 60 kV were used for the acquisition of the data.

A scanning electron microscope (SEM) was used for fractography of CCF filament on Phenom Pro X (Thermo Fisher Scientific, Waltham, MA, USA), using secondary electrons at 15 kV.

## 3. Result and Discussion

### 3.1. Fractography

**Figure 5:**
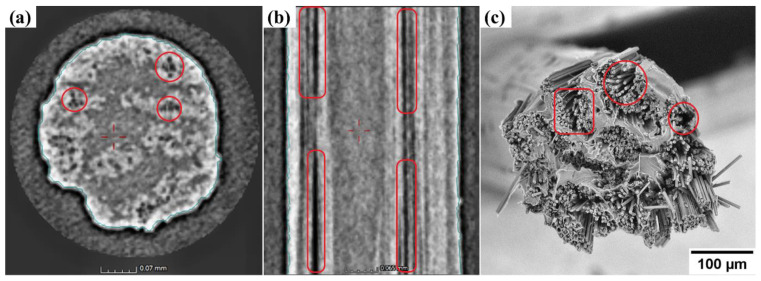
Images acquired via CT scan and SEM. Overview of CCF via CT (**a**); sectional view of CCF via CT (**b**); cryofractured surface of CCF via SEM (**c**).

**Figure 6:**
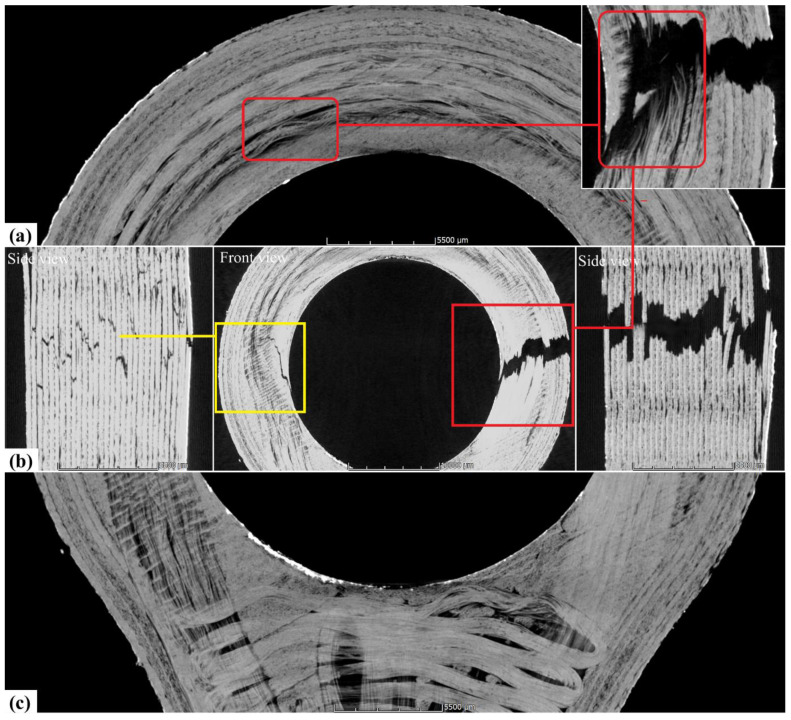
CT scan of the fractured lug with an overview of two fractured zones. Maximum principal stress direction CCF layup with a primary fractured zone as an inset (**a**); overview and side views of primary (red frame) and secondary (yellow frame) fractures (**b**); minimum principal stress direction CCF layup (**c**). The red connecting line indicates the cause and effect in the fractured region (undulation and voids).

A CT scan of fractured lug specimen and cryofractured CCF was performed to investigate the fracture behaviour of the additively manufactured layered composite and to identify the inherent defects within the CCF. In Figure 5a, analysis of the CT image of CCF assists in identifying the bright region as carbon fibre bundles, light greyscale region as the thermoplastic zone and dark spots highlighted within red circles as voids. Similarly, Figure 5b is a CT sectional image of the CCF with sections of voids highlighted within the red capsules. The quantifiable void content within the CCF was found to be approximately 1.5 vol.%. In Figure 5c, the SEM image of cryofractured CCF validates the detection of voids within the CCF in CT scans. The loose carbon fibres (red box and circle) observed are due to low infusion of highly viscous PA6 thermoplastic matrix between individual fibres causing fibre slippage and entrapping air bubbles within the CCF. This is detrimental to the overall performance of CCF, as the capability of the carbon fibre bundle to carry strain uniformly is diminished. Single carbon fibre fracture results in early unpredictable composite failures, as highlighted in the inset of Figure 6a (red box). Furthermore, the process-induced voids are also a cause for drastic influence in forming undulation in CCF layup shown in Figure 6a (red box), mainly as a result of buckling of loose carbon fibres during CCF extrusion and in situ consolidation due to nozzle compaction, similarly reviewed by Sanei and Popescu [28].

Figure 6a shows a single layer maximum principal direction layup along with a layered composite primary fracture zone CT image as an inset. Figure 6b shows the overview and primary and secondary fractures as the side view of the tensile-tested lug. The primary fracture is indicated by the red frame, and the secondary fracture is represented within the yellow frame. The CCF layers break within a narrow window around the location of the maximum stress, indicating some strength variation over the length of the CCF and potentially undulation-induced load variation between layers highlighted in Figure 6a (red box), and the reasoning is that already discussed in Figure 5. In Figure 6b, the CT scan of both primary and secondary fractures shows uneven failure, which is an attribute of MEX specimens similarly reported by authors Savandaiah et al. [3] and Spoerk et al. [5]. Voids between each layer are inherent to MEX due to layered processing and result in poor load transfer between layers, causing jagged teeth fractured appearance [4,17]. Furthermore, in Figure 6b side views, the CT scan shows some delamination, which may have occurred during fracture of single layers; however, this cannot be resolved from the postfracture examination data. In Figure 6c, the minimum principal direction layup and the twisted CCF bundles around the curved sections are shown. According to the authors Shiratori et al. [29], twisting due to curving caused the early damage to fibres during the CCF layup, and the process-induced voids within the broken and twisted CCF may negatively influence the overall mechanical performance of the MEX specimens [30]. Videos of the CT scan data are provided in the supplementary data in Videos S1 and S2 for front and side views, respectively.

### 3.2. FEA and Strain Trajectory Analysis

**Figure 7:**
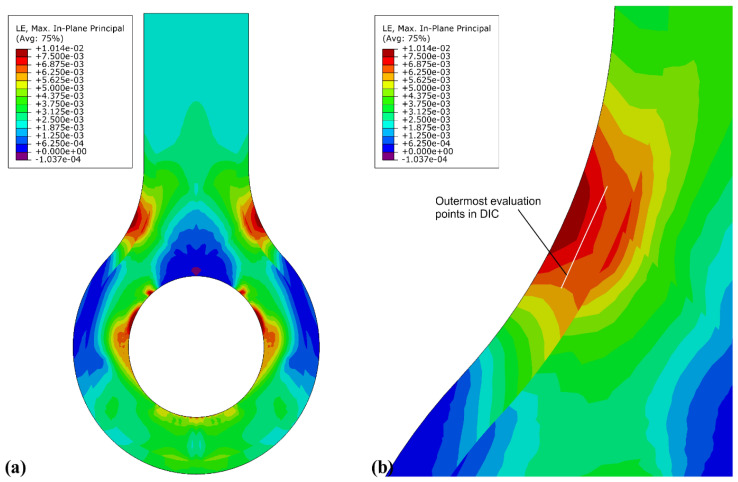
Maximum principal strains on the orthotropic model under tensile load. Full lug (**a**); detailed neck area where the DIC evaluation area limit is indicated (**b**).

**Figure 8:**
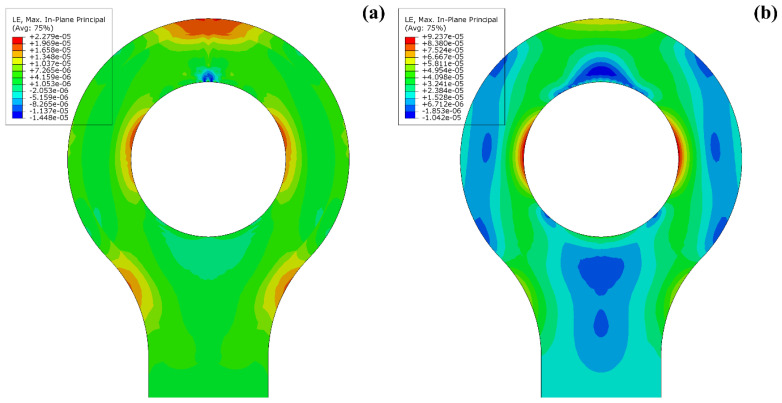
Maximum principal strains for unit tensile load on the lugs. Short carbon fibre reinforced PA6 quasi-isotropic material (**a**); load-path curvilinear continuous carbon fibre reinforced material (**b**). The significant reduction in strain due to the fibre reinforcement is clearly visible. Additionally, the superior strength of the CCF material may be fully utilised.

Figure 7 gives strain results for the plane-strain model with a tensile line load of 1300 N·mm^−1^ applied to the shaft of the lug which is reacted in the lug eye. This load was chosen for comparison to measurement data later in this paper. The maximum principal strain in the neck region is ε_1,max,neck_ = 8.10 × 10 ^−3^, and at the outermost point for which the DIC can obtain values, 1 mm inside the edge for this analysis, the strain is ε_1,DIC,neck_ = 7.23 × 10^−3^. These strains are in the perimeter region where only short-fibre-reinforced thermoplastic material was printed. The absolute maximum of principal strains is ε_1,max_ = 8.50 × 10^−3^ at the lug eye, where CCF was printed. Because of the modulus difference between thermoplastic and CCF material, the stresses are much larger at the lug eye than at the neck. However, the area around the lug eye was obstructed from view in testing by the test fixture, and no strain measurement was taken here. In the shaft (top), away from the strain concentrations, a quasi-uniaxial strain of ε_1,shaft_ = 2.35 × 10^−3^ was obtained. The values are compared to measured strains in Table 2. The quasi-isotropic (see Figure 8) and the curvilinear orthotropic designs were all predicted to fail due to fibre tension near the lug eye. Though the curvilinear design had the same failure mode, it achieved the design consideration of transferring the applied load effectively around the eye. The strain near the lug eye was highest in quasi-isotropic design, in contrast to the material near curvilinear design.

### 3.3. Evaluation of Modelling by Experimental Testing

**Figure 9:**
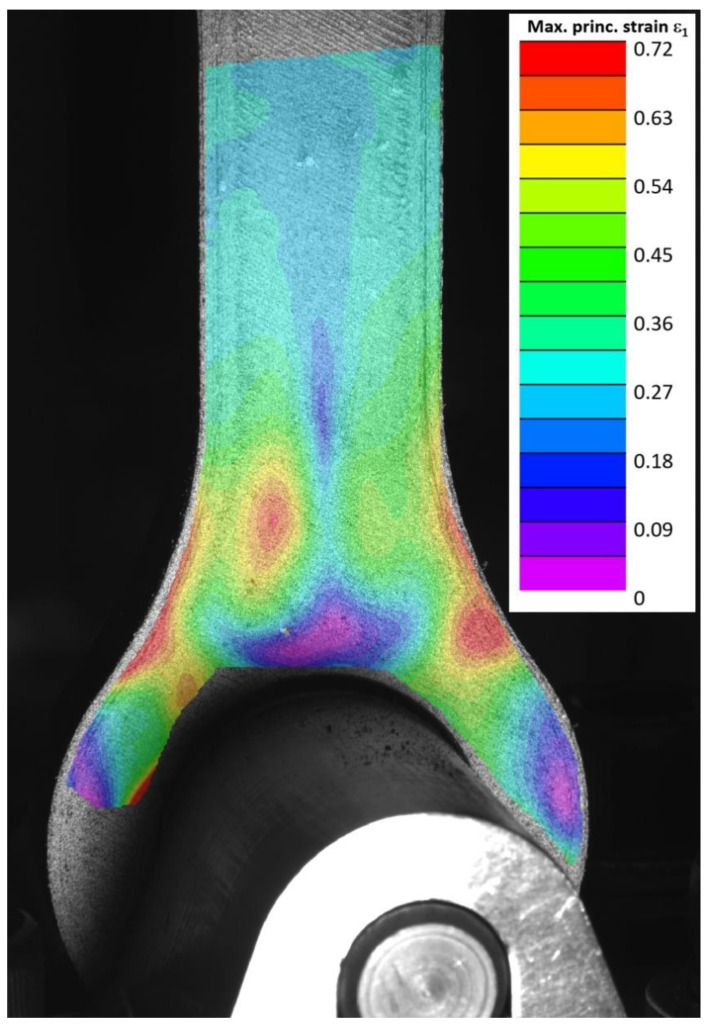
Major principal strain in tested lug No. 2.

**Figure 10:**
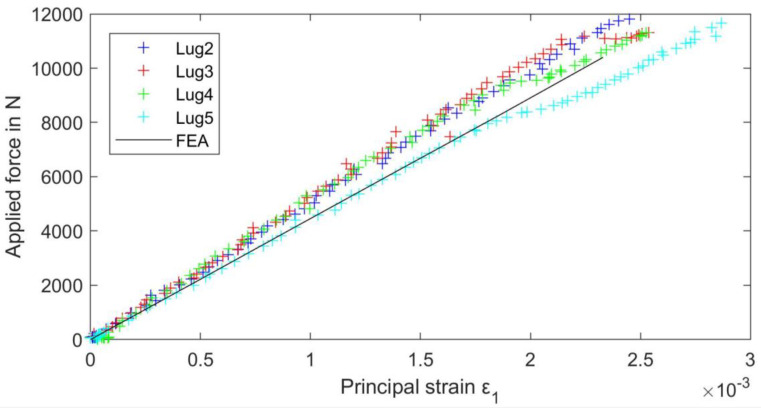
Force over maximum principal strains for the lug shaft from testing.

In Figure 9, the image clearly shows the loading conditions of the tested lug and the strain variations. The obtained FE results were compared against experimental test results, in which surface strains were monitored by optical DIC measurement. Because of the mounting of the lug, not all of the surface was visible to the DIC. However, high strain areas at the neck of the lug were clearly in view. The test specimen lug No. 2 provided excellent DIC patterns and insight into material behaviour. Figure 9 shows the major principal strain obtained in testing at a load of 11,590 N. The DIC evaluation allows strains to be measured only a finite distance from the part boundary, and in the case of the lug this is about 1 mm. Note the similarity in the strain distribution compared to Figure 7. This strain pattern also makes specimens more susceptible to failures due to MEX defects reported earlier causing premature failure of the specimen due to the strain being concentrated in the individual CCF layers. This phenomenon can be observed in Figure 10 as variation in graph noise for the tested specimen.

To indicate the level of agreement between FE and test results, maximum principal strains at the centre of the shaft and the strain concentration on the neck were compared for similar shaft strain levels, and the strain concentration factor was calculated as a ratio of neck to shaft strains. The corresponding values are given in Table 2.

In addition to the strain levels, the stiffness and strength of the lug were also evaluated. As was shown earlier, the fracture occurred at the lug eye, perpendicular to the load, and unfortunately, the DIC measurement could not capture this area because of the rig setup. To estimate the stresses in this area of the part at failure, FE results were used. Maximum principal stresses σ_max,FE_ in the lug head were obtained for a load equivalent to the average maximum force from experimental tests and are reported in Table 3. Because the 2D FEA reports smeared results only, the stress values from FEA were corrected to reflect that only every other layer of the material in the lug head is CCF-reinforced. This value can be compared to the MEX composite coupons tested according to ASTM D3039 [31], and data derived from the supplier for fibre strength of the CCF material gives good agreement.

The strain at high load, shown in Figure 7 from nonlinear FEA, shows that compared to lower loads, e.g., in Figure 8, the maximum strain at the eye shifts slightly towards the shaft end. The fractured specimens in Figure 6 show the fracture line at the eye in a similar position. The fracture initiation of the printed part coincides with the numerically highest loaded area, giving confidence in the FEA.

Figure 10 shows the applied force on the test rig over the DIC-obtained maximum principal strain in the shaft of the lug. For comparison, FEA-obtained shaft strain over applied force is included. The data for tensile strength and a linear approximation of the part stiffness are presented in Table 4. There is little variability in the strength, and the fracture initiates at the location of maximum stress for all tests. The stiffness is similar for all tested lugs, averaging at 4.59 με·N^−1^, and agrees well with the FEA results, 4.46 με·N^−1^.

Generally, the results obtained for the AM lug show that the FEA-assisted design of fibre trajectories can predict the mechanical part properties well. The part stiffness and strain distribution as well as the failure location and strength could be predetermined to a good degree. The methodology can be easily applied to different AM structures, and it is reasonable to believe that equally good results are achievable in terms of structural stiffness and strain distribution. For part failure determination under more complex loading conditions, a suitable multiaxial composite failure criterion has to be employed.

## 4. Conclusions

The potential of using additively manufactured CCF parts as load-carrying lugs has been presented in this study. The part design was based on FEA and gave load-defined fibre trajectories. Experimental tests with the printed parts were performed.

The main finding of this investigation is that FE analysis data and experimental test results agreed well in terms of stiffness and strain. The FE-calculated part stiffness was higher by a factor of 1.03, and the strain distributions were very similar. The strain concentration around the neck differed by only 1%. Therefore, the potential of using CAE-aided fibre steering for the design of additively manufactured CCF parts is given. A further conclusion is that, potentially because there is some inherent variability in the manufacturing process and in the material, a strength reduction of 8% in the manufactured lugs compared to the manufacturer material data is yielded. As an application guideline, a correction factor should be considered in engineering design to compensate for this.

In the present part, a viable fibre layup in line with the principal axis was obtained in one analysis step. In future work, including analysis of more complicated part geometries, the analysis may have to be performed in optimisation loops until the obtained principal material axis deviates less than a certain set value from the obtained principal stress or strain axis. Furthermore, limits on fibre orientation change over length, i.e., a minimum radius of the fibre bundle, may have to be considered when performing more extensive optimisation on complex geometries. Lastly, geometry optimisation was not part of the objectives of the present study but may be a future addition to the method.

## Figures and Tables

**Table 1:** Detailed printing settings for CCF and FFF print head.

Print Settings	Unit	Value
Layer height	mm	0.15
CCF layer width	mm	1
FFF layer width	mm	0.2
Nozzle diameter	mm	0.7
Lug thickness	mm	8.5
CCF print head temperature	°C	250
FFF print head temperature	°C	260
Print bed temperature	°C	95
CCF print speed (red)	mm·min^−1^	550
CCF print speed (green)	mm·min^−1^	400
FFF print speed	mm·min^−1^	2200
Maximum principal stress layers (CCF and FFF print head)	-	29
Minimum principal stress layers (CCF and FFF print head)	-	27
Top plastic layer (FFF print head)	-	1

**Table 2:** Comparison of FE and experimental test results: maximum strain around the neck compared to strain at shaft.

Position	FE	DIC
Shaft (ε_1,shaft_)	0.00235	0.00245
Neck (ε_1,DIC,neck_)	0.00723	0.0073 (avg. left and right)
Strain concentration factor	3.08	3.02

**Table 3:** Comparison of FE maximum principal stresses and Markforged data.

Value	FE Max. Principal Stress	CCF Strength
Absolute	734 MPa	800 MPa [32]
Relative	0.92	1.00

**Table 4:** Strength and stiffness of the lugs from testing.

Tested Lugs	Strength (kN)	Linear Stiffness (με·N^−1^)
Lug 2	11.81	4.93
Lug 3	11.32	4.77
Lug 4	11.32	4.64
Lug 5	11.66	4.03
Average	11.53	4.59
FEA	-	4.46

## Data Availability

Further data is available on request from the authors.

## Supplementary Materials

The following supporting information can be downloaded at: https://www.mdpi.com/article/10.3390/ma15051820/s1, Figure S1: Prirevo CCF filament used in the manufacturing of the lugs; Video S1: Video of CT Scan of fractured specimen, front view; Video S2: Video of CT Scan of fractured specimen, side view.
