# Supplementary figures and images for: Additively Manufactured Composite Lug with Continuous Carbon Fibre Steering Based on Finite Element Analysis
Source: Materials (Basel). 2022 Feb 28;15(5):1820. doi: 10.3390/ma15051820 (PMC8911614; doi:10.3390/ma15051820)

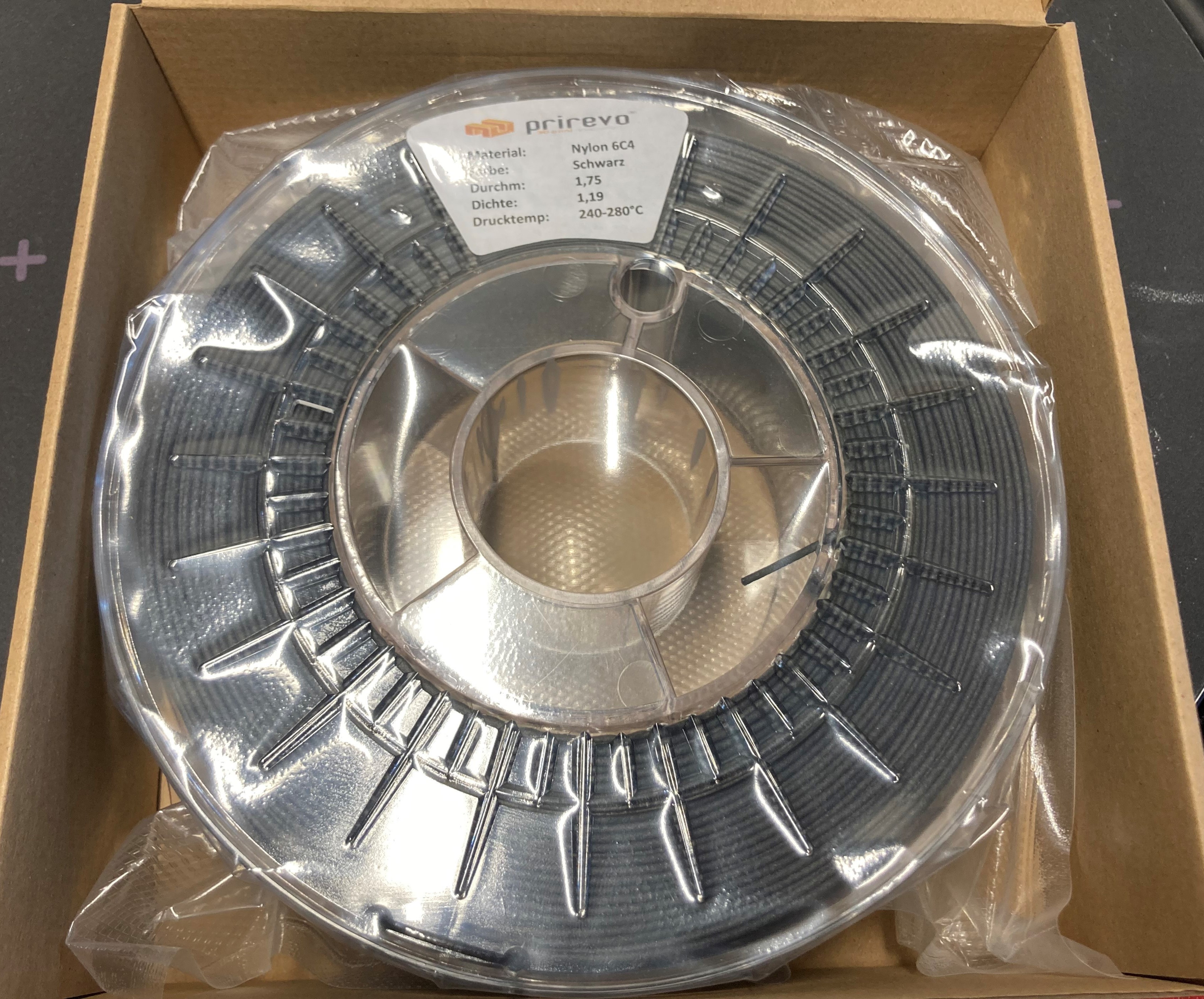

Supplement: Supplementary file 1 [file materials-15-01820-s001.zip › Figure S1.jpg]
